# Exposure to environmental pharmaceuticals affects the macromolecular composition of mussels digestive glands

**DOI:** 10.1038/s41598-024-59663-7

**Published:** 2024-04-23

**Authors:** Marica Mezzelani, Valentina Notarstefano, Michela Panni, Elisabetta Giorgini, Stefania Gorbi, Francesco Regoli

**Affiliations:** 1 Dipartimento di Scienze della Vita e dell’Ambiente (DiSVA), Università Politecnica delle Marche, Ancona, 60131 Italy; 2 NBFC, National Biodiversity Future Center, Palermo, 90131, Italy

**Keywords:** Environmental pharmaceuticals, Carbamazepine, Mechanisms of action, Lipid metabolism, Digestive gland, *Mytilus galloprovincialis*, Infrared spectroscopy, Biological models, Experimental organisms, Model invertebrates, Marine biology

## Abstract

Human pharmaceuticals represent a major challenge in natural environment. A better knowledge on their mechanisms of action and adverse effects on cellular pathways is fundamental to predict long-term consequences for marine wildlife. The FTIRI Imaging (FTIRI) spectroscopy represents a vibrational technique allowing to map specific areas of non-homogeneous biological samples, providing a unique biochemical and ultrastructural fingerprint of the tissue. In this study, FTIRI technique has been applied, for the first time, to characterize (i) the chemical building blocks of digestive glands of *Mytilus galloprovincialis*, (ii) alterations and (iii) resilience of macromolecular composition, after a 14-days exposure to 0.5 µg/L of carbamazepine (CBZ), valsartan (VAL) and their mixture, followed by a 14-days recovery period. Spectral features of mussels digestive glands provided insights on composition and topographical distribution of main groups of biological macromolecules, such as proteins, lipids, and glycosylated compounds. Pharmaceuticals caused an increase in the total amount of protein and a significant decrease of lipids levels. Changes in macromolecular features reflected the modulation of specific molecular and biochemical pathways thus supporting our knowledge on mechanisms of action of such emerging pollutants. Overall, the applied approach could represent an added value within integrated strategies for the effects-based evaluation of environmental contaminants.

## Introduction

Pharmaceuticals represent emerging pollutants for aquatic environments, commonly found and accumulated by non-target organisms, affecting several biochemical and physiological processes with virtually unknown long-term effects^1–4^. The Mediterranean mussel, *Mytilus galloprovincialis* is particularly sensitive to pharmaceutical pollution and many laboratory and field studies highlighted the capability of these organisms to accumulate large variety of active principles, such as carbamazepine (CBZ), valsartan (VAL), paroxetine (PAR), lormetazepam (LOR), diclofenac (DIC), and ibuprofen (IBU)^5^. Under laboratory conditions, environmentally realistic levels of these compounds affected both early life stages and adult mussels, leading, to an evident impairment of immune, oxidative and lipid metabolisms^6–9^: these effects have been mainly measured in terms of changes in gene expression, transcriptional profile, modulation of several enzymes representative of specific pathways, accumulation of peroxidation products, alteration of physiological traits (e.g. reduction of byssus abundance and strength), and impairment of feeding behaviour. Such findings allow to hypothesise that modulation of specific metabolic pathways by pharmaceuticals might cause possible alterations in the macromolecular composition of target tissues in terms of physical and structural changes of biomolecules such as lipids, proteins, and carbohydrates.

The Fourier Transformed InfraRed Imaging (FTIRI) represents a powerful vibrational technique, widely applied in human biomedicine to study the macromolecular building blocks and composition of cells and tissues. The coupling of IR spectrometers with bidimensional arrays detectors enables to spectroscopically map specific areas of non-homogeneous biological samples, providing a unique biochemical and ultrastructural fingerprint of the tissue. Characterization of the most of relevant molecules typically present in a biological sample (i.e. proteins, lipids, sugars and nucleic acids) can be directly related to specific pathways and processes, such as cellular activity, activation of metabolisms and oxidative pathways^10–16^. For this reason, FTIRI spectroscopy is largely applied in clinical studies to characterize structural properties in normal and pathological samples, enabling the rapid diagnosis for many diseases like endocrinological disturbances, tumoral lesions and inflammations^13,14,17^. In this respect, ROS-related neurotoxicity of amphetamine has been proved to cause the onset of biochemical alterations of lipids and proteins in brain sections of albino rats^18^. Raman spectroscopy for cancer diagnosis has been reviewed^19^, summarizing the suitability of this technique in the detection of small biomolecular changes typically associated with cancer such as an increased nucleus-to-cytoplasm ratio, disordered chromatin, higher metabolic activity, and changes in lipid and protein levels. The FTIRI characterization of polysaccharides, nucleic acids, collagen, amides, lipids, proteins, and fatty acids in oral mucosa revealed the effects of inflammatory fibrous hyperplasia, providing important molecular information to be used as a marker of ongoing inflammatory processes. A specific set of spectral biomarkers mainly related to lipid and carbohydrate metabolism, and to cell transcriptional activity has been recently identified for discriminating the two main typologies of pancreatic neoplasms^16^. In aquatic species, FTIRI spectroscopy was also recently applied for detecting early changes in lipid content and fatty acids modifications in response to several feed additives and pollutants in zebrafish^20^, while data on marine organisms are still lacking.

In this respect the present study was aimed to (i) characterize for the first time the biochemical composition and structure of *M. galloprovincialis* digestive gland by FTIRI, defining its specific macromolecular fingerprint; (ii) investigate the onset of spectral alterations in response to pharmaceuticals to better elucidate mechanisms of actions and (iii) to validate the use of a similar approach when assessing cellular effects of emerging pollutants.

This study was carried out on mussels exposed to the antiepileptic carbamazepine (0.5 µg/L), the antihypertensive valsartan (0.5 µg/L) and their mixture (0.5 µg/L for each drug). After 14 days of exposure, organisms were maintained for additional 14 days in carbamazepine- and valsartan-free artificial seawater, intended as recovery phase from the tested active pharmaceuticals ingredients.

The exposure doses of CBZ and VAL are environmentally realistic and typically found in coastal areas^21–24^. The rationale for selecting 14 days for both the exposure and depuration phase was based on previous knowledge on physiology and responsiveness of the selected bioindicator species to different typologies of environmental pollutants^25–28^. These organisms have been previously analysed in terms of variations of gene expression, impairment of neurotransmission, cell cycle, immune responses, and redox homeostasis and these results were useful for a more comprehensive functional interpretation of actual FTIRI findings^6^. Overall the present study was expected to clarify whether changes in macromolecular features of tissues can be related to the onset of molecular and cellular effects caused by pharmaceuticals, thus enhancing our knowledge on mechanisms of action and risk of such emerging pollutants.

## Results

### Spectral characterization and macromolecular composition of *M. galloprovincialis* digestive gland

**Figure 1:**
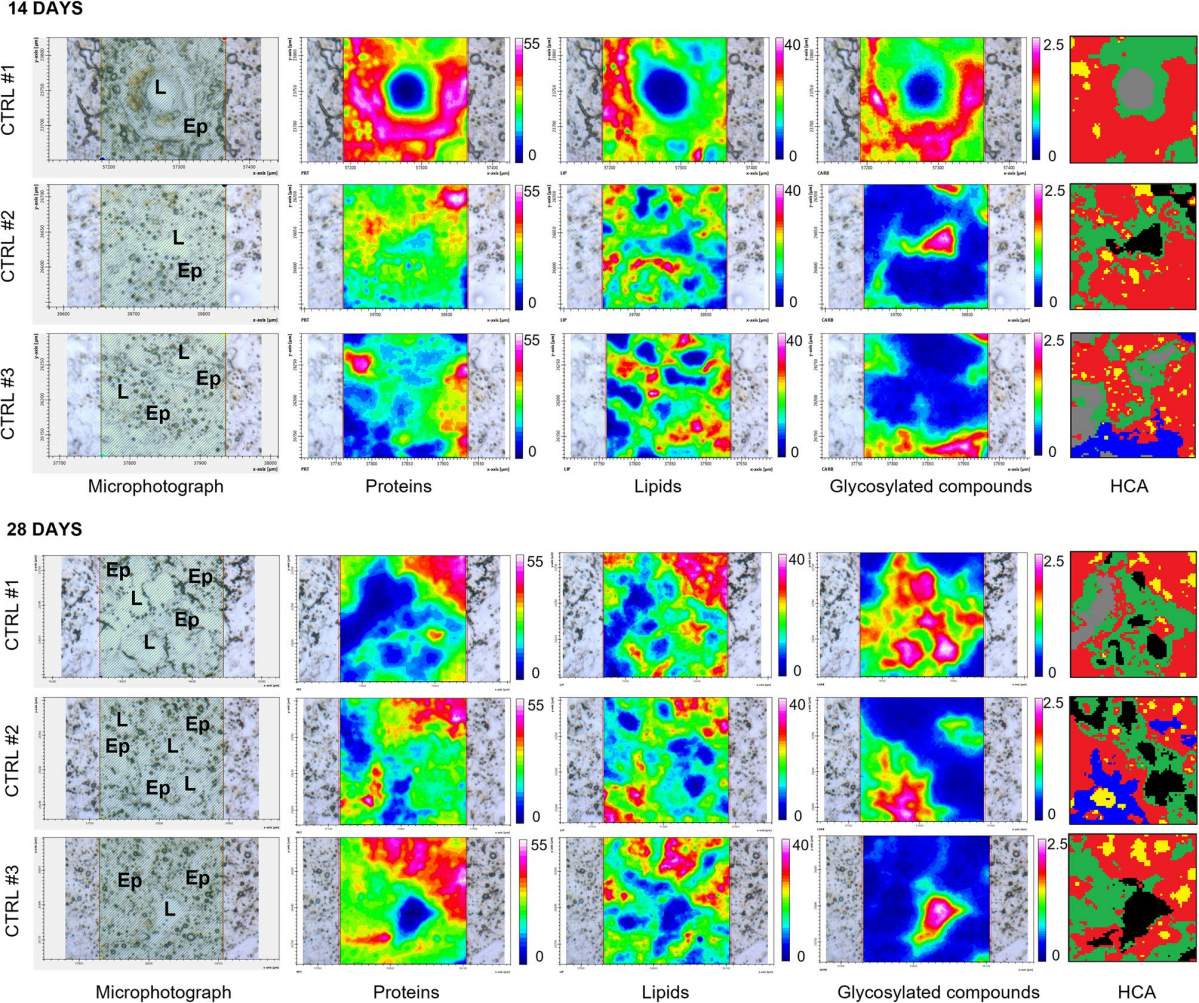
Black and white microphotographs, hyperspectral analysis and HCA cluster map of representative mussels digestive gland sections belonging to Control groups at 14 and 28 days. False colour imaging shows the topographical distribution of proteins, lipids and glycosylated compounds. The comparison between each black and white microphotograph with associated HCA cluster map allow to characterize main components of digestive tissue and to discriminate different histological areas of tubules, mainly the lumen (L) from the epithelium (Ep). The spectral characterization (HCA) highlights: in grey scale semi-quantitative differences in terms of amount of glycosylated compounds present in the lumen (light grey corresponds to empty lumen, dark grey to medium, and black to higher levels of glycosylated compounds); in green the inner epithelium; in red the outer epithelium; in yellow the lipid-rich regions and in blue random spots of glycosylated compounds.

Microphotographs, hyperspectral imaging analyses, and HCA cluster maps of digestive glands of Control mussels are shown in Fig. 1; three replicates are displayed, representative of different areas and histological structures. A detailed description of representative tissues sections is reported in Supplementary Information S1 (SI), along with additional IR images for each unstained section of all experimental groups. The hyperspectral imaging analyses generated three false colours images indicating the topographical distribution of proteins, lipids and glycosylated compounds, respectively. By considering the different scales adopted for each macromolecule, digestive glands of Control groups, at both 14 and 28 days, were characterized by a higher amount of proteins (PRT images, numerical scale 0–55) compared to lipids (LIP images, numerical scale 0–40) and glycosylated compounds (GLY images, numerical scale 0–2.5). The comparison between each microphotograph with associated HCA cluster map enabled the discrimination of different histological areas, mainly the lumen (L) from the epithelium (Ep). The spectral characterization revealed glycosylated compounds as the predominant typology of macromolecules occurring inside the lumen; semi-quantitative differences in terms of amount of such compounds are highlighted in the HCA cluster maps in grey scale: light grey corresponds to empty lumen, dark grey to medium, and black to higher levels of glycosylated compounds. FTIRI analyses revealed that the area associated to the epithelium (Ep) of digestive tubules is constituted by two regions characterized by a specific and unique spectral fingerprint. Although false colour images reveal that both are mainly constituted by proteins, the first region, coloured in green in HCA cluster maps, is smaller and closer to the lumen, while the other one, in red, is larger and covers the majority of tubule’s surface. For better clarity, the first area was here defined as inner epithelium, while the second as outer epithelium. In the latter it was also possible to observe particularly lipid-rich regions, highlighted in yellow in HCA cluster maps. Random spots of glycosylated compounds, not associated with specific areas of the epithelium were also detected outside the lumen and are highlighted in blue.

**Figure 2:**
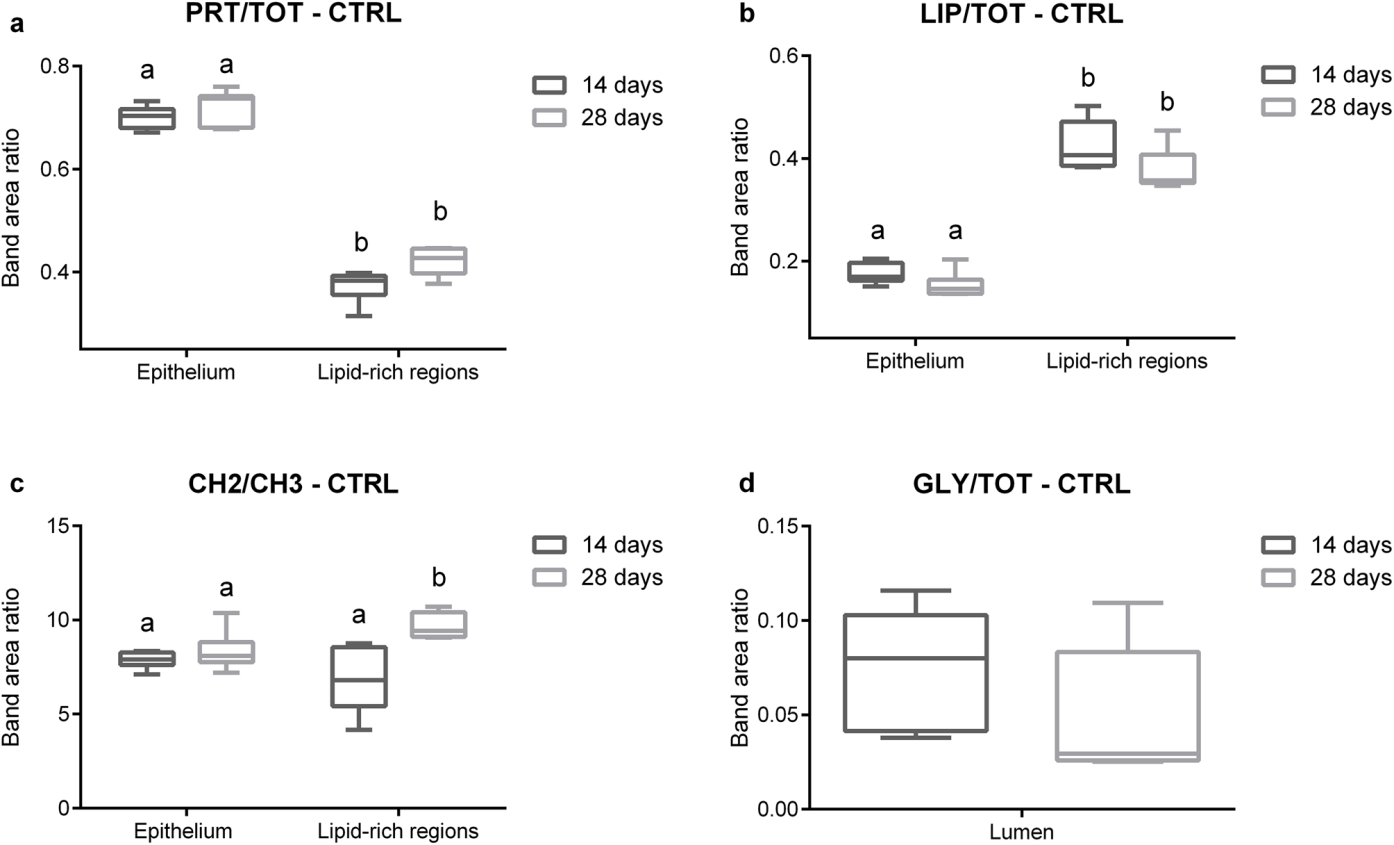
Biochemical composition of representative digestive gland sections. Box plots show the values of the PRT/TOT (**a**), LIP/TOT (**b**), CH2/CH3 (**c**), and GLY/TOT (**d**) band area ratios calculated in the epithelia, lipid-rich regions, and lumina of Control samples collected at 14 and 28 days: centre line marks the median, edges indicate the 25th and 75th percentile, whiskers indicate the min and max values. Different letters indicate a statistically significant difference between groups. Statistical significance was set at 0.05 and calculated by Student’s *t*-test.

The biochemical composition of all Control mussels digestive glands at day 14 and 28 (Fig. 2) was then measured and compared by univariate analysis in terms of specific band area ratios representing the relative amount of proteins (PRT/TOT), lipids (LIP/TOT), length of alkyl chains (CH2/CH3, index of lipid saturation) in the overall epithelium and in lipid rich regions; the relative amount of glycosylated compounds (GLY/TOT) was assessed inside the lumen. In the outer epithelium the presence of lipid-rich areas revealed by HCA cluster maps (Fig. 1) was confirmed by the semi-quantitative analyses of PRT/TOT and LIP/TOT band area ratio, which highlighted statistically significant lower levels of proteins and higher levels of lipids in such areas compared to rest of the epithelium (Fig. 2a,b).

In all Control samples no significant variation between 14 and 28 days was observed in terms of semi-quantitative characterization of proteins (Fig. 2a) and lipids (Fig. 2b) in the epithelium and lipid-rich regions. Significant increment in levels of saturation (Fig. 2c) was measured in lipid-rich regions at 28 days compared to 14, while no differences between the two sampling periods occurred in terms of the amount of glycosylated compounds inside the lumen (Fig. 2d). Overall, the lack of significant differences between 14 and 28 days for most of the analysed parameters indicated high spectral homogeneity of qualitative and quantitative macromolecules composition in mussels digestive glands, allowing to use FTIRI technique in revealing effects caused by tested pharmaceuticals in *M. galloprovincialis*.

### Biological effects of cabamazepine, valsartan and their mixture on mussels digestive gland macromolecular composition

**Figure 3:**
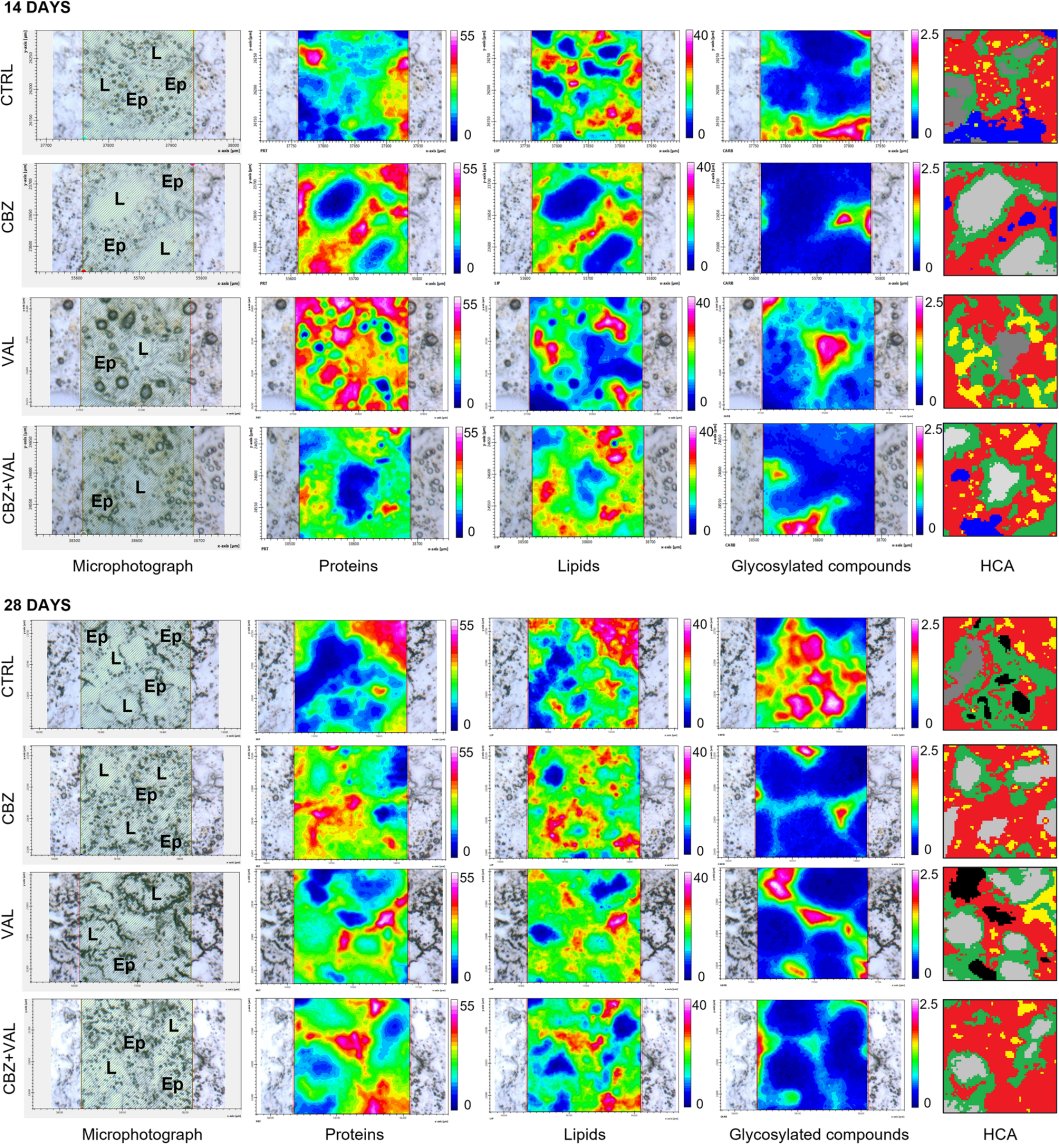
Black and white microphotographs, and hyperspectral analysis of representative mussels digestive gland sections belonging to all treatments (Control, CBZ, VAL and CBZ + VAL) at 14 and 28 days. False colour imaging shows the topographical distribution of proteins, lipids and glycosylated compounds. Different colour scales were used for a better interpretation of the data. The comparison between each black and white microphotograph with associated HCA cluster map allow to characterize main components of digestive tissue and to discriminate different histological areas of tubules, mainly the lumen (L) from the epithelium (Ep). The spectral characterization (HCA) highlights: in grey scale semi-quantitative differences in terms of amount of glycosylated compounds present in the lumen (light grey corresponds to empty lumen, dark grey to medium, and black to higher levels of glycosylated compounds); in green the inner epithelium; in red the outer epithelium; in yellow the lipid-rich regions and in blue random spots of glycosylated compounds.

**Figure 4:**
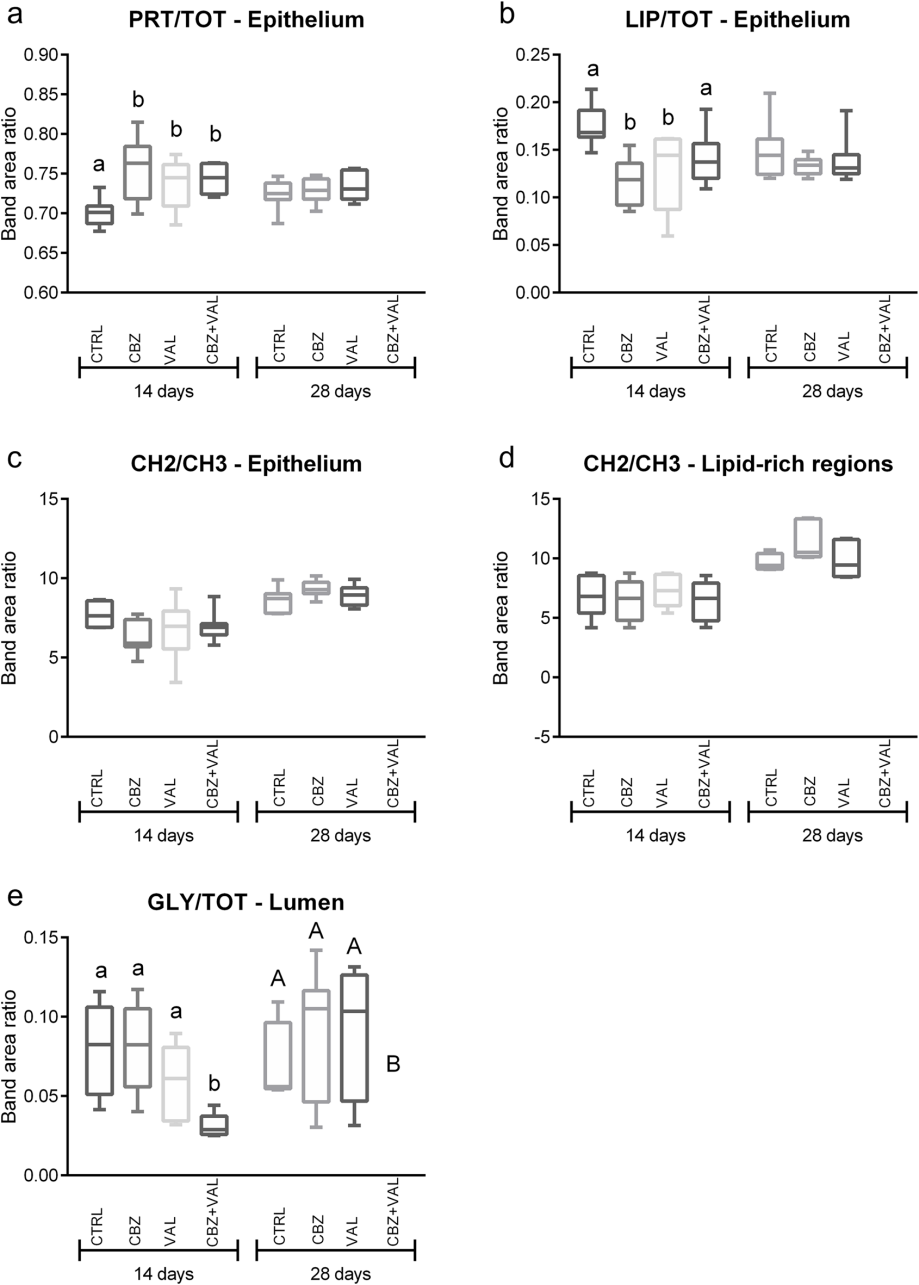
Biochemical composition of representative digestive gland sections. Box plots show the values of: the PRT/TOT (**a**), LIP/TOT (**b**), CH2/CH3 (**c**) band area ratio of the epithelium; the CH2/CH3 of the lipid rich- regions (**d**); and the GLY/TOT band area ratio of the lumen regions (**e**). Samples are divided according to the treatment and to the time of collection. Centre line marks the median, edges indicate the 25th and 75th percentile, whiskers indicate the min and max values. Different letters indicate a statistically significant difference between Control and each of the other groups, separately at 14 days and 28 days. Statistical significance was set at 0.05, and calculated by One-way ANOVA, followed by Dunnett’s multiple comparison test.

Figure 3 shows microphotographs, hyperspectral analyses, and HCA cluster maps of representative sections of mussels digestive gland after 14 and 28 of various treatments (CTRL, CBZ, VAL and CBZ + VAL). Also for these samples, a detailed description of sections and additional IR images are given for all experimental groups in Supplementary Information S1 (SI). The hyperspectral imaging analyses generated three false colours images showing the topographical distribution of proteins, lipids, and glycosylated compounds. Considering the different scales adopted for each macromolecule, digestive glands from all the treatments, at both 14 and 28 days, were always characterized by a higher amount of proteins (PRT images, numerical scale 0–55) compared to lipids (LIP images, numerical scale 0–40) and glycosylated compounds (GLY images, numerical scale 0–2.5). Concerning the effects of the exposures to CBZ, VAL and CBZ + VAL, the FTIR images indicated an increment of proteins and a decrease of lipids after 14 days-exposure; the lumina of CBZ + VAL samples appeared to contain a minor amount of glycosylated compounds. After the end of the depuration phase, limited differences among treatments were observed. These effects were confirmed by the composition of specific band area ratios representative of the relative amount of proteins (PRT/TOT), lipids (LIP/TOT), length of alkyl chains (CH2/CH3) in the overall epithelium, length of alkyl chains in lipid-rich regions and the relative amount of glycosylated compounds inside the lumen (GLY/TOT) (Fig. 4). No variations in terms of PRT/TOT and LIP/TOT band area ratios were found within the lipid-rich regions in all treatments at 14 and 28 days (data not shown). After 14 days of exposure, a significant increase in proteins levels was measured in mussels treated with CBZ, VAL and CBZ + VAL compared to the control, while no changes were observed at the end of depuration phase (Fig. 4a). Lowered amounts of lipids was observed in all treatments after 14 days of exposure, with a sharp and significant decrease in mussels exposed to CBZ and VAL alone; at the end of the recovery period no differences were measured between Control and exposed mussels (Fig. 4b). A lack of significant changes was reported in terms of CH2/CH3 in the epithelium, after both 14 and 28 days (Fig. 4c), while higher mean levels in length of alkyl chains were measured in all samples at the end of the recovery period compared to 14-days treatments (Fig. 4d). A significant decrease in the amount of glycosylated compounds in mussels lumen was observed for the mixture treatment both at the end of exposure and depuration period (Fig. 4e).

## Discussion

Environmental consequences of pharmaceuticals have recently emerged as a major threat affecting natural ecosystems. Ongoing changes of the climate system and interactions in complex mixtures may further influence the possible impacts of pharmaceuticals with unexpected effects, highlighting the urgency of modern approaches for effect-based assessment of adverse biological outcomes. The Mediterranean mussels, *Mytilus galloprovincialis*, is a key sentinel species due to its high ecological value, commercial interest and the sensitivity of molecular and cellular responses toward environmental stressors. Digestive glands represent the main target tissue for chemicals uptake and metabolization, thus being intensively investigated for estimating the impacts of such stressors^26^. In the present study, the application of FTIRI spectroscopy allowed for the first time, to characterize composition and topographical distribution of proteins, lipids and glycosylated compounds, integrating new functional data with histological and histochemical knowledge of morphological structures and typologies of cells^29,30^. A specific pattern characterized the cluster of alveoli of all analysed mussels, with a clear discrimination between two main areas of the epithelium: one smaller and closer to the lumen, reported here as inner epithelium, enclosed by the outer epithelium covering most of the tubule’s surface. Although both these areas are mainly constituted by proteins, each of them is characterized by a unique spectral signature, possibly associated to diverse cell composition and function elicited by these two regions. Digestive gland is the target tissue for nutrient absorption, intracellular digestion and storage of reserves, and its single epithelium is composed by digestive and basophilic cells: the first are eosinophilic due to the highly developed endo-lysosomal system responsible for the intracellular digestion of food materials, while basophilic cells contain a rough endoplasmic reticulum, numerous secretory granules and are mainly involved in the synthesis and secretion of hydrolytic enzymes^30^. Despite digestive cells are more likely localized toward the lumen and the basophilic in the basal region of digestive gland^31^, many studies demonstrated changes in this pattern according to mussels reproductive period and exposure to stressful conditions, thus reflecting a more heterogeneous distribution and lack of clear separation within the epithelium surface^30,32^. Digestive cells are characterized by a canal system while basophilic cells have secretory granules; notably, both are involved in endocytic processes and therefore connected to the lumen when they finally secrete the digested content. From our results it might be speculated that the inner epithelium concentrates digestion-phase related cells, that release their digested content into the lumen. Previous studies on bivalves digestive tubules demonstrated that they typically undergo three cyclic phases (absorption, digestion and fragmentation/excretion), lacking of uniform appearance due to non-synchronous digestive processes related to their feeding behaviour which is considered to be continuous^33^.

Studies on functional histology and ultrastructure of *M. galloprovincialis* digestive glands extensively documented the presence of lipid-rich regions in epithelium^34–36^, with lipid droplets typically interspersed throughout the cytoplasm of digestive cells^35^. In the present study, FTIRI spectroscopy clearly discriminated such lipid-rich areas mainly occurring in the outer epithelium, allowing to hypothesize this area as mainly involved in nutrient absorption and reserve storage (absorption-phase related cells).

Among lipids, fatty acids have been documented as the most abundant in mussels digestive glands, with both polar and neutral lipids, and saturated forms as the dominant groups^37–39^. In the present study, the vibrational imaging analysis allowed to characterize the profile of saturated lipids (by means of CH2/CH3 band area ratio). Those variations reflect the activation of lipid peroxidation processes (changes in the intracellular formation of reactive oxygen species) and changes due to the reproductive cycle (i.e. gametogenesis)^37,38^. Furthermore, such lipids provide energy to cope with different typologies of environmental stressors and sudden changes in their content are useful in revealing early warning signals of disturbance^36,40^.

No investigations have been previously focussed on qualitative and quantitative assessment of the lumen content in mussels’ digestive gland. In the present study, FTIRI spectroscopy allowed to characterize glycosylated compounds as the main molecules occurring inside the lumen of mussels tubules. Besides being the major secondary metabolites of plant cells, thus reflecting ingredients of mussels diet, glycosylated compounds can results from proteins synthesis and cellular turnover. In this respect, it is possible to associate such molecules to digestive cell debris excreted to the lumen from the digestive alveus, which have been demonstrated to have a lysosomal origin^41^.

Fluctuation in the amount of glycosylated compounds in lumina of the same organism confirmed the above-mentioned non-synchronous digestive processes. Noteworthy, FTIRI spectroscopy allowed to highlight that glycosylated compounds are also present in spot, random areas of the epithelium or connective tissue, consistent with previous observations on the storage of digestion products in dedicated vesicles of the connective tissue and absorptive cells of the epithelium^34,42^. In general, this study demonstrated a high homogeneity in results obtained by FTIRI on control mussels as demonstrated by the lack of significant variation in all analysed parameters between days 14 and 28. Such findings suggest the potential of vibrational imaging analysis as additional and integrative approach to measure biochemical, functional, and ultrastructural effects caused by environmental stressors.

In this respect, changes in macromolecular profile were investigated in exposed mussels to assess whether they reflect mechanism of action and onset of adverse effects caused by environmental pharmaceuticals like carbamazepine and valsartan^27,43,44^: beside CBZ constant release in the aquatic environment, this molecule is highly resistant to degradation processes with reported half-life in surface waters ranging from 38 to 1200 days (3.5 years)^45,46^. Conversely, no information can be found on VAL persistence in aquatic environments.

The interactive effects of the antiepileptic carbamazepine and the antihypertensive valsartan have been recently characterized through the analyses of large number of responses including bioaccumulation, changes in transcriptional profile and measurements on biochemical and cellular alterations^6^. The complex network of observed responses highlighted significant variations of gene expression, functional effects on neurotransmission, cell cycle, immune responses, redox homeostasis, with a greater biological reactivity of CBZ and the onset of antagonistic effects when in mixture with VAL.

When the same samples were analysed through vibrational imaging analysis, a significant increase in the amount of proteins was observed in all treatments after 14 days of exposure, with particularly higher levels in mussels exposed to single compounds compared to the mixture. These results are consistent with those obtained in our previous study^6^, allowing a functional interpretation of FTIRI results and to speculate on possible mechanisms and processes behind such findings: future metabolomic studies might further validate proposed hypothesis. Both pharmaceuticals caused significant transcriptional changes in genes related to cell cycle and cellular turnover, closely related to protein metabolism^47^. For CBZ, results obtained in the present work, support the evidence of the cell cycle stimulation though the Akt, MAPK, PI3K-FRAP/mTOR pathways, as a MOA-related effect of this antiepileptic compound toward proliferation and survival processes^48^.

For VAL, the involvement of protein metabolism might similarly being connected with its mechanism of action, since the angiotensin II receptor was found to increase the phosphorylation of endothelial NO synthase (eNOS) via Src/PI3K/Akt signalling pathways^49^. In our previous study^6^, CBZ determined activation of redox metabolism with significant transcriptional modulation of genes encoding phase I and phase II related proteins, and heat shock proteins (CYP1A1, SULT1C4, SACS, HSP12B and HSP12A), with the parallel catalytic induction of most of the antioxidant enzymes. Similarly, the marked induction of the biotransformation enzyme glutathione S-transferases (GST) in all treatments, and the enhancement in VAL-exposed mussels of ubiquitin-protein transferase regulatory activity and protein turnover^6^, further supported the increased proteins levels observed here by FTIRI after the end of the exposure phase (14 days). Interestingly, the application of infrared microspectroscopy in mammals demonstrated a strong linear correlation between the levels of proteins and ROS^18^. Results obtained in the present study with a significant enhancement of proteins in mussels exposed to CBZ, VAL and their mixture are in agreement with the modulation of oxidative pathways^6^: at the same time, the lack of significant variation in terms of levels of lipid saturation after 14-days exposure (by means of CH2/CH3 band area ratio) is consistent with the reported lack of increment in levels of lipofuscin in mussels digestive glands^6^.

The significant decrease of lipids highlighted by FTIRI in all treatments is also concomitant with a significant inhibition of the Acyl CoA oxidase (ACOX) activity previously reported in exposed mussels^6^: catalysing the first reaction of β- oxidation of fatty acids, ACOX promotes lipid degradation, and it is thus possible to hypothesise that this mechanism is lowered to avoid excessive reduction of these important energy reserves in stress conditions as a strategy to maintain cellular lipid homeostasis. For VAL this result is particularly relevant since it is consistent with the therapeutic activity of this drug in target species as regulator of glucose-and lipid metabolism, capable of decreasing levels of cholesterol, possibly due to the link between the angiotensin II receptor type 1-dependent and peroxisome proliferator-activated receptors γ (PPAR γ) signaling pathways^50^. Considering the key role of lipids in mussels physiology, changes in lipid profile measured through FTIRI, demonstrate the progression of effects measured at molecular and biochemical level up to cellular and physiological levels in response to pharmaceuticals. In mussels exposed to the CBZ-VAL mixture, the significantly lower amount of glycosylated compounds in the lumina of digestive tubules would suggest some kind of synergism in the modulation of digestive processes, contrasting with the antagonist interaction previously reported for the effects of these two molecules^6^.

In conclusion, the present study represents a significant progress in the comprehensive understanding of topographical distribution of the main macromolecules namely, proteins, lipids and glycosylated compounds in *M. galloprovincialis* digestive gland. Similar results had never been reported in marine organisms exposed to pharmaceuticals, allowing to demonstrate different changes in mussels exposed to various experimental conditions. The integration of such new functional data further extends our capability to measure alterations caused by human pharmaceuticals in non-target marine species. For the first time carbamazepine, valsartan and their mixture showed to clearly modulate the overall amount of proteins and lipids consistent with the modulation of molecular and biochemical pathways previously measured on common samples. Finally, the study of macromolecular features and the application of similar approach in invertebrates could also have a broader perspective in the development of new effect-based methodologies, for the early detection of biological effects of environmental stressors.

## Methods

### Animal collection, experimental design and sample preparation

Mussels, *Mytilus galloprovincialis* (5.3 ± 0.5 cm shell length), were obtained in June 2020 from a shellfish farm in a reference area of central Adriatic Sea^17^. The late spring is considered as one of the post-spawning periods of the year *for M. galloprovincialis* in this area^40^. Collection and experimental use of mussels is not subjected to ethical review permissions according to both European and Italian normative (Directive 2010/63/EU, 2010; Italian Legislative Decree n. 26, 4/03/2014). Acclimation and exposure conditions have been detailed elsewhere^6^. Briefly, organisms were acclimatized for 10 days with aerated artificial seawater (ASW) at local seasonal temperature (23 °C), salinity (35 practical salinity units), and pH (8.20), then randomly assigned, without sex-ratio assessment, to eight 20 L tanks, each containing 60 organisms, and exposed to one of the following treatments, performed in duplicate: CTRL, control condition; CBZ, carbamazepine exposure (0.5 µg/L); VAL, valsartan exposure (0.5 µg/L); CBZ + VAL, combined mixture of carbamazepine (0.5 µg/L) and valsartan (0.5 µg/L). After 14 days of exposure (exposure phase), organisms were allowed to depurate for additional 14 days in carbamazepine- and valsartan-free ASW (depuration phase). The exposure doses of CBZ and VAL are environmentally realistic and typically found in coastal areas^21,22,24^. Stock solutions of carbamazepine and valsartan (Sigma Aldrich) were prepared in methanol and stored at room temperature for the duration of the experiment, while working solutions were prepared daily by diluting the stock solution in ASW. Water was changed and pharmaceuticals re-dosed every other day; mussels were fed 12 h prior the water change with 500 μL of a commercial mixture of phytoplankton (EasyBooster PRO) for filter-feeding organisms, according to manufacturer indications. From each experimental condition, organisms were sampled at day 14 and 28 (i.e. 14 days of exposure + 14 days in carbamazepine- and valsartan-free ASW): 10 digestive glands were rapidly dissected, separately frozen in liquid nitrogen and maintained at − 80 °C. All the samples were cut by a cryostat and 10 sections (8 µm thick) for each digestive gland were deposited onto CaF2 optical windows (1-mm thick, 13-mm diameter) and let air-dry for 30 min without any fixation process. FTIRI measurements were performed within 48 h after cutting; this procedure was already carried out on similar samples and a good stability in terms of infrared features was always observed^12,16,17^.

### FTIRI measurements and data analysis

The spectral imaging analysis enables to map specific areas of non-homogeneous biological samples, generating false colour images, that represent the topographical distribution of the total absorption of infrared radiation. Each pixel corresponds to an IR spectrum, and the intensity of the signal associated with a specific IR band provides information both on the amount and the localization of the corresponding molecular/chemical groups^12^. In the present study, for each unstained section of all experimental groups, 7 IR images were acquired in transmission mode (15X condenser/objective; 4000–900 cm^−1^; 4 cm^−1^ spectral resolution, and 256 scans) on specific regions of interest. Background spectra were obtained on clean regions of the CaF2 optical windows with the same acquisition parameters. Raw IR images were corrected with Atmospheric Compensation and Baseline correction (two-points linear fitting) routines (OPUS 7.5 software, Bruker Optics GmbH, Ettlingen, Germany). False colour images were generated by integrating IR images in specific spectral regions, to evaluate the spatial distribution and the total amount of proteins (1720–1480 cm^−1^, Amide I and II bands, vibrational modes of peptide linkage), lipids (2995–2828 cm^−1^, vibrational modes of lipid alkyl chains), and glycosylated compounds (1065–1010 cm^−1^, vibrational modes of C-O groups in carbohydrates and glycosylated compounds). Hierarchical Cluster Analysis (HCA) of the IR images was performed using CytoSpec 2.00.01 software (CytoSpec, Inc., Berlin, Germany), by Euclidean distances and Ward’s linkage method, for classifying spectra based on their profile and relate them to specific histological structures. For each cluster identified by HCA, spectra were extracted and submitted to the integration procedure (integration Mode B, OPUS 7.5 software): 2995–2950 cm^−1^ (symmetric and asymmetric stretching modes of CH3 moieties of lipid alkyl chains, CH3), 2948–2828 cm^−1^ (asymmetric stretching mode of CH2 groups in alkyl chains, CH2), 2995–2828 cm^−1^ (CH2 and CH3 groups in lipid alkyl chains, LIP),1720–1480 cm^−1^ (vibrational modes of peptide linkage, PRT), and 1065–1010 cm^−1^ (stretching mode of C-O moieties in carbohydrates and glycosylated compounds, GLY). The following band area ratios representative of the relative amount of proteins (PRT/TOT), lipids, (LIP/TOT), lipid saturation (CH2/CH3) and glycosylated compounds (GLY/TOT) were then calculated, by using the abovementioned integrated areas. Total tissue biomass (TOT) was calculated by the sum of the integrated areas in the regions 3031–2828 cm^−1^ and 1781–980 cm^−1^.

### Statistical analyses

The spectral characterization of Control digestive gland sections was subjected to univariate analysis by means of a Student’s *t*-test (statistical difference was set at p < 0.05; Prism6, Graphpad software, Inc., San Diego, CA, USA), to assess the possible changes of the macromolecular composition from 14 to 28 days, with no treatment.

For the validation of spectral modification as a biomarker of pharmaceuticals exposure, univariate analysis was performed by means of a factorial analysis of variance (one-way ANOVA), followed by Tukey’s multiple comparison test (Prism6, Graphpad software, Inc., San Diego, CA, USA). One-way ANOVA was used to compare the means of Control, CBZ, VAL, and CBZ + VAL at 14 days and of Control, CBZ, VAL, and CBZ + VAL at 28 days, to make inferences about the population means. Statistical significance was set at p < 0.05.

### Supplementary Information

Supplementary Information.

## Data Availability

The data that support the findings of this study are available on request from the corresponding author FR.
