# Supplementary material for: Exposure to environmental pharmaceuticals affects the macromolecular composition of mussels digestive glands
Source: Sci Rep. 2024 Apr 23;14:9369. doi: 10.1038/s41598-024-59663-7 (PMC11039728; doi:10.1038/s41598-024-59663-7)

**Exposure to environmental pharmaceuticals affects the macromolecular composition of mussels digestive glands.**

^1^Marica Mezzelani, ^1^Valentina Notarstefano, ^1^Michela Panni, ^1^Elisabetta Giorgini, ^1-2^Stefania Gorbi, and ^1-2^Francesco Regoli*

^1^Dipartimento di Scienze della Vita e dell’Ambiente, Università Politecnica delle Marche, via Brecce Bianche (60131), Ancona, Italy

^2^ NFBC, National Future Biodiversity Center, Palermo, Italy

**Supplementary Information**

***Description of components of two representative tissues sections***

Mussels’ digestive gland sections of Control organism (Fig. S1 a-b) is characterized by one digestive tubule covering the majority of microphotograph surface, with a small round-shaped lumen (L) surrounded by the digestive tubule’s epithelium (Ep).

Digestive gland sections of CBZ-exposed mussel (Fig. SI 1 c-d) is characterized by part of two digestive tubules, the first one on the top, covering the majority of microphotograph surface, while a second on the bottom. Two large round-shaped lumina (L) are shown, surrounded by the digestive tubule’s epithelium (Ep).

**Figure S1**

Microphotographs of mussels’ digestive gland sections of Control (a) and CBZ-exposed (c) organisms collected after 14-days exposure. Edited microphotographs (b-d), highlight lumen (L) and digestive tubule’s epithelium (Ep).

*
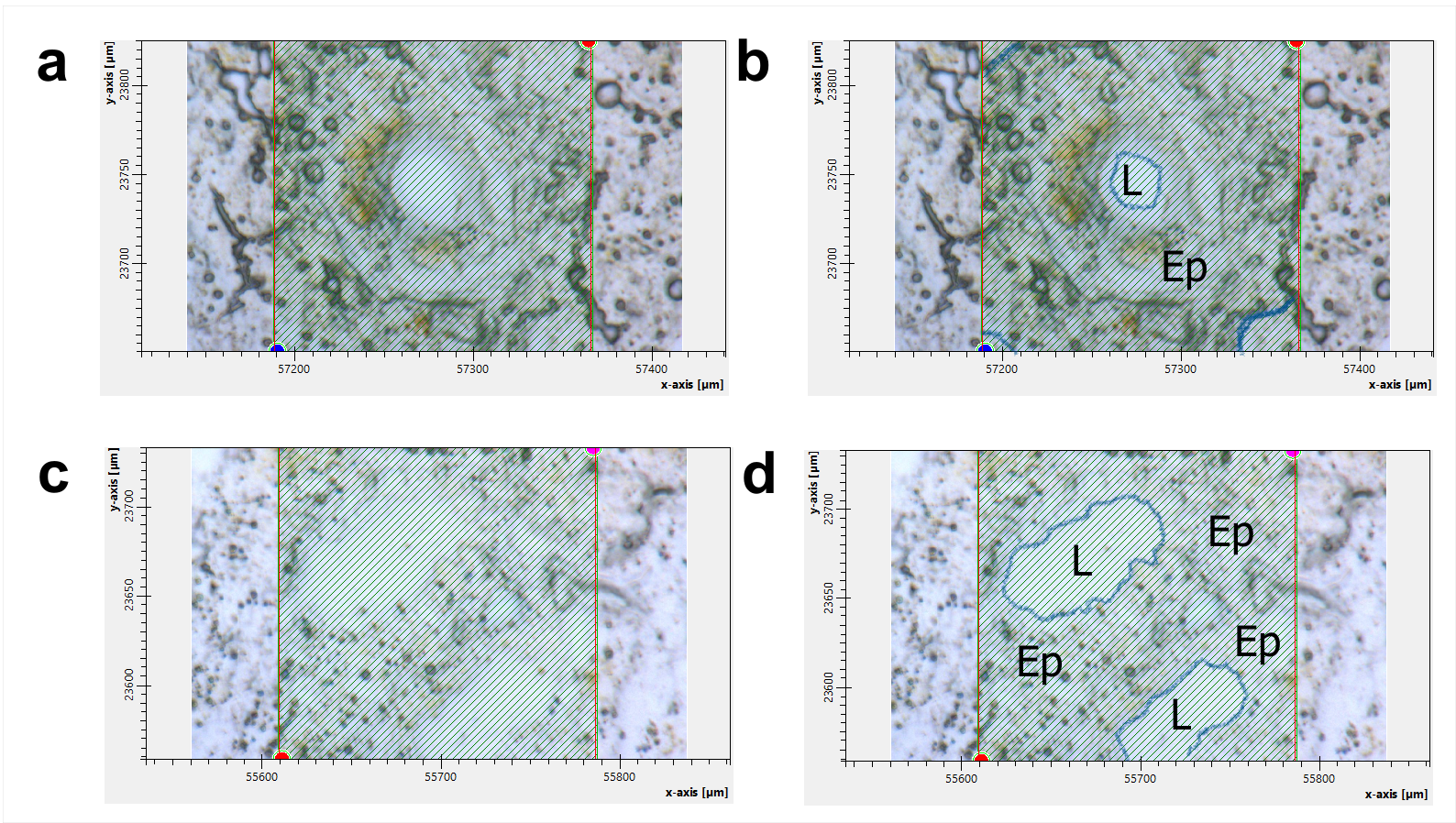
*

***FTIRI measurements and data analysis.***

Four additional IR images for each unstained section of all experimental groups acquired in transmission mode (15X condenser/objective; 4000-900 cm-1; 4 cm-1 spectral resolution, and 256 scans) on specific regions of interest not showed in the main MS file.

**Figure S2**

Black and white microphotographs, hyperspectral analysis and HCA cluster map of representative mussels digestive gland sections belonging to Control group at 14 days. False colour imaging shows the topographical distribution of proteins, lipids and glycosylated compounds. The comparison between each black and white microphotograph with associated HCA cluster map allow to characterize main components of digestive tissue and to discriminate different histological areas of tubules, mainly the lumen from the epithelium. The spectral characterization (HCA) highlights: in grey scale semi-quantitative differences in terms of amount of glycosylated compounds present in the lumen (light grey corresponds to empty lumen, dark grey to medium, and black to higher levels of glycosylated compounds); in green the inner epithelium; in red the outer epithelium; in yellow the lipid-rich regions and in blue random spots of glycosylated compounds.


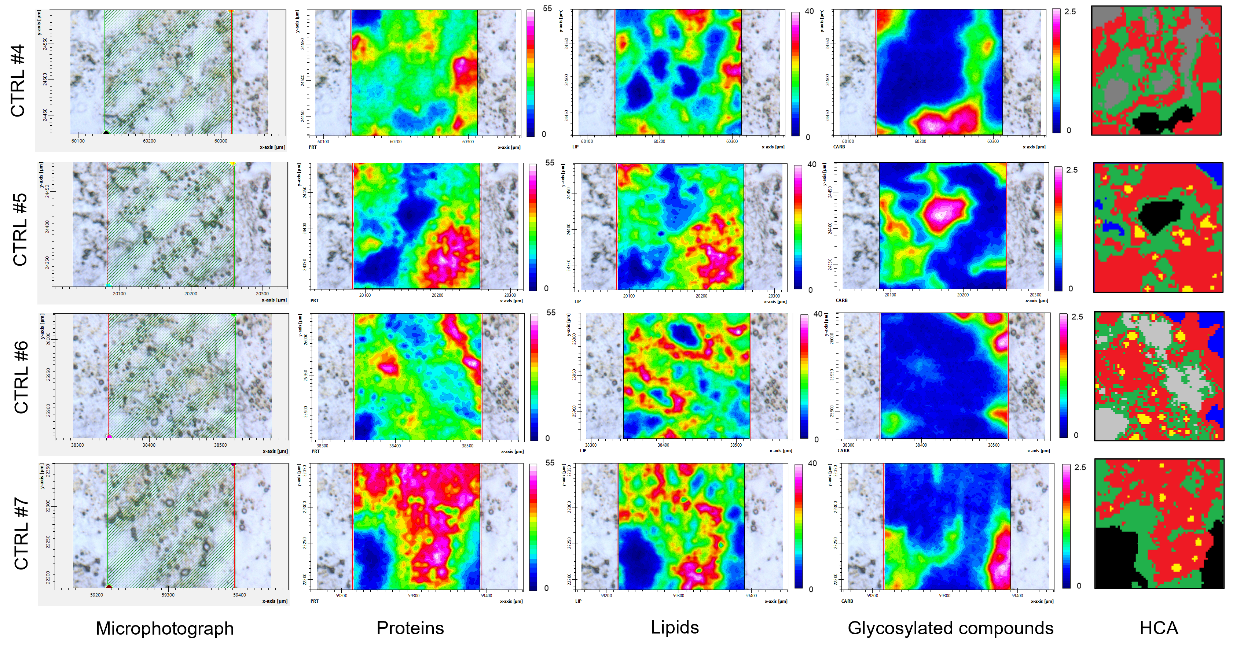


**Figure S3**

Black and white microphotographs, hyperspectral analysis and HCA cluster map of representative mussels digestive gland sections belonging to Control group at 28 days. False colour imaging shows the topographical distribution of proteins, lipids and glycosylated compounds. The comparison between each black and white microphotograph with associated HCA cluster map allow to characterize main components of digestive tissue and to discriminate different histological areas of tubules, mainly the lumen from the epithelium. The spectral characterization (HCA) highlights: in grey scale semi-quantitative differences in terms of amount of glycosylated compounds present in the lumen (light grey corresponds to empty lumen, dark grey to medium, and black to higher levels of glycosylated compounds); in green the inner epithelium; in red the outer epithelium; in yellow the lipid-rich regions and in blue random spots of glycosylated compounds.


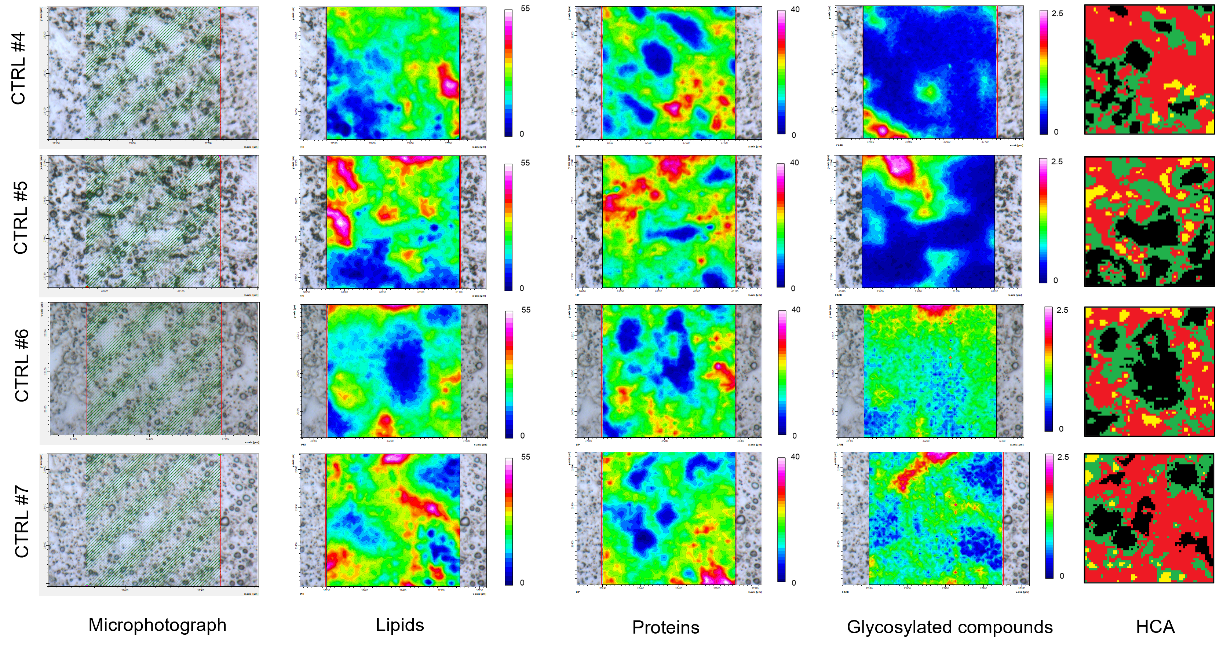


**Figure S4**

Black and white microphotographs, hyperspectral analysis and HCA cluster map of representative mussels digestive gland sections belonging to CBZ group at 14 days. False colour imaging shows the topographical distribution of proteins, lipids and glycosylated compounds. The comparison between each black and white microphotograph with associated HCA cluster map allow to characterize main components of digestive tissue and to discriminate different histological areas of tubules, mainly the lumen from the epithelium. The spectral characterization (HCA) highlights: in grey scale semi-quantitative differences in terms of amount of glycosylated compounds present in the lumen (light grey corresponds to empty lumen, dark grey to medium, and black to higher levels of glycosylated compounds); in green the inner epithelium; in red the outer epithelium; in yellow the lipid-rich regions and in blue random spots of glycosylated compounds.


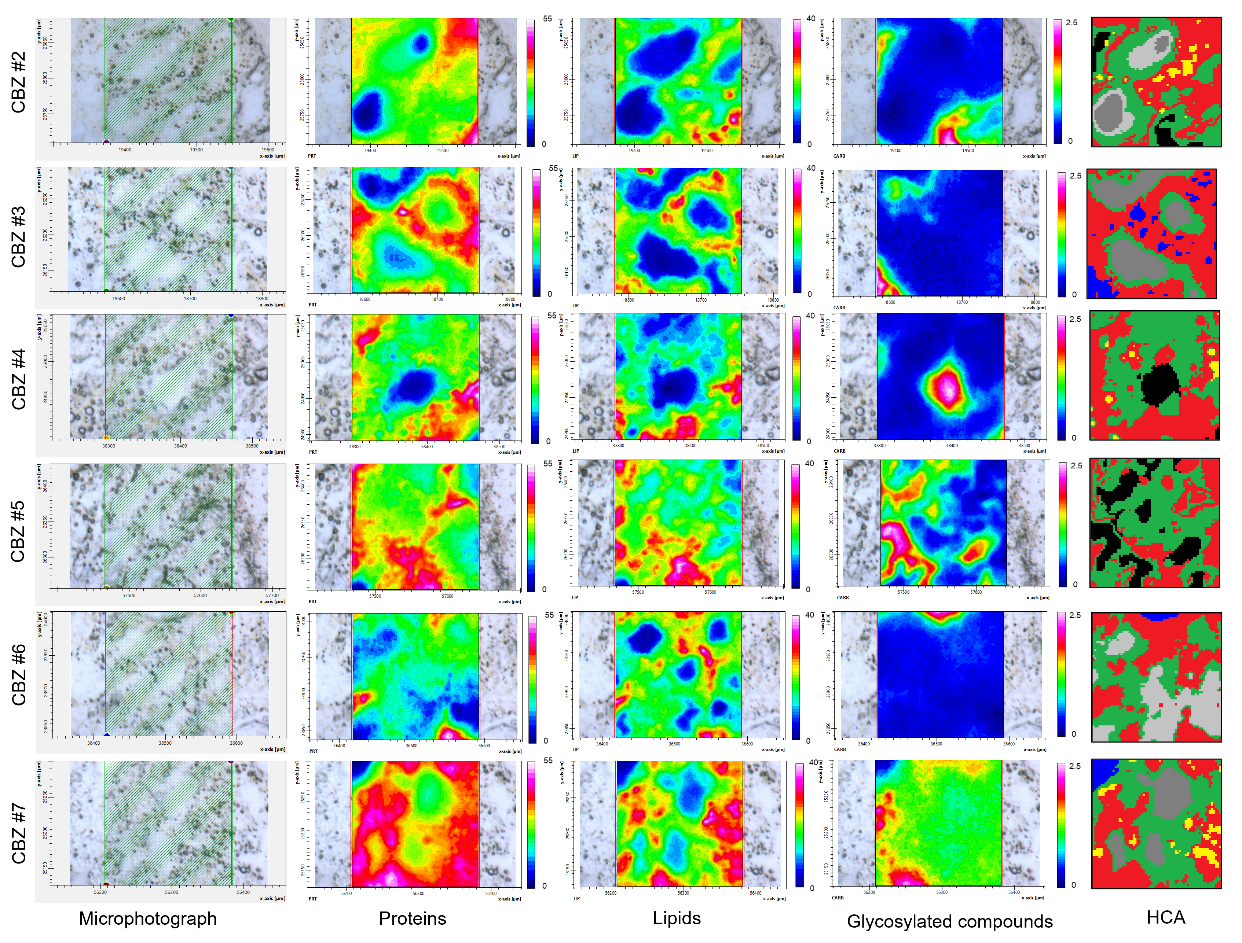


**Figure S5**

Black and white microphotographs, hyperspectral analysis and HCA cluster map of representative mussels digestive gland sections belonging to CBZ group at 28 days. False colour imaging shows the topographical distribution of proteins, lipids and glycosylated compounds. The comparison between each black and white microphotograph with associated HCA cluster map allow to characterize main components of digestive tissue and to discriminate different histological areas of tubules, mainly the lumen from the epithelium. The spectral characterization (HCA) highlights: in grey scale semi-quantitative differences in terms of amount of glycosylated compounds present in the lumen (light grey corresponds to empty lumen, dark grey to medium, and black to higher levels of glycosylated compounds); in green the inner epithelium; in red the outer epithelium; in yellow the lipid-rich regions and in blue random spots of glycosylated compounds.


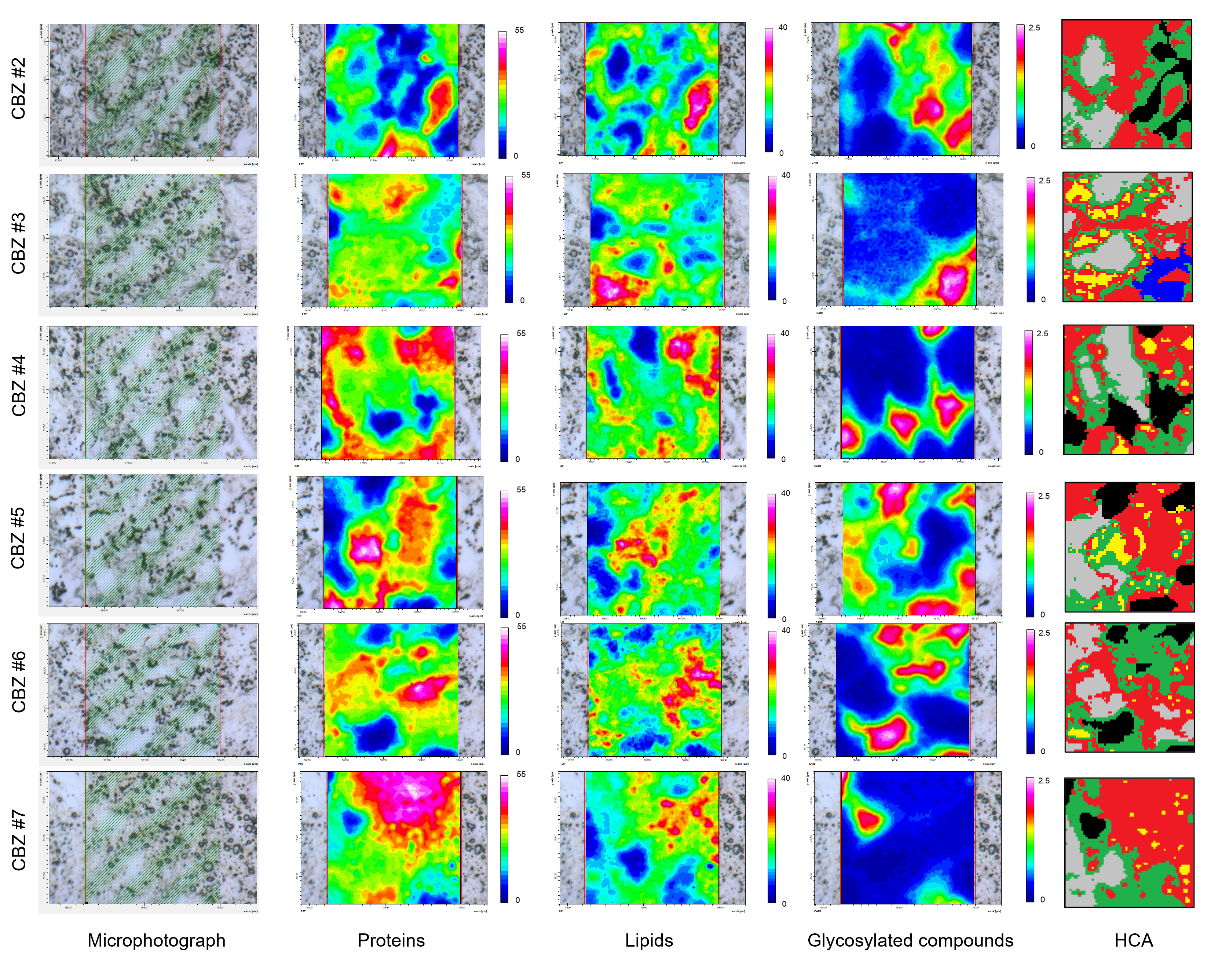


**Figure S6**

Black and white microphotographs, hyperspectral analysis and HCA cluster map of representative mussels digestive gland sections belonging to VAL group at 14 days. False colour imaging shows the topographical distribution of proteins, lipids and glycosylated compounds. The comparison between each black and white microphotograph with associated HCA cluster map allow to characterize main components of digestive tissue and to discriminate different histological areas of tubules, mainly the lumen from the epithelium. The spectral characterization (HCA) highlights: in grey scale semi-quantitative differences in terms of amount of glycosylated compounds present in the lumen (light grey corresponds to empty lumen, dark grey to medium, and black to higher levels of glycosylated compounds); in green the inner epithelium; in red the outer epithelium; in yellow the lipid-rich regions and in blue random spots of glycosylated compounds.


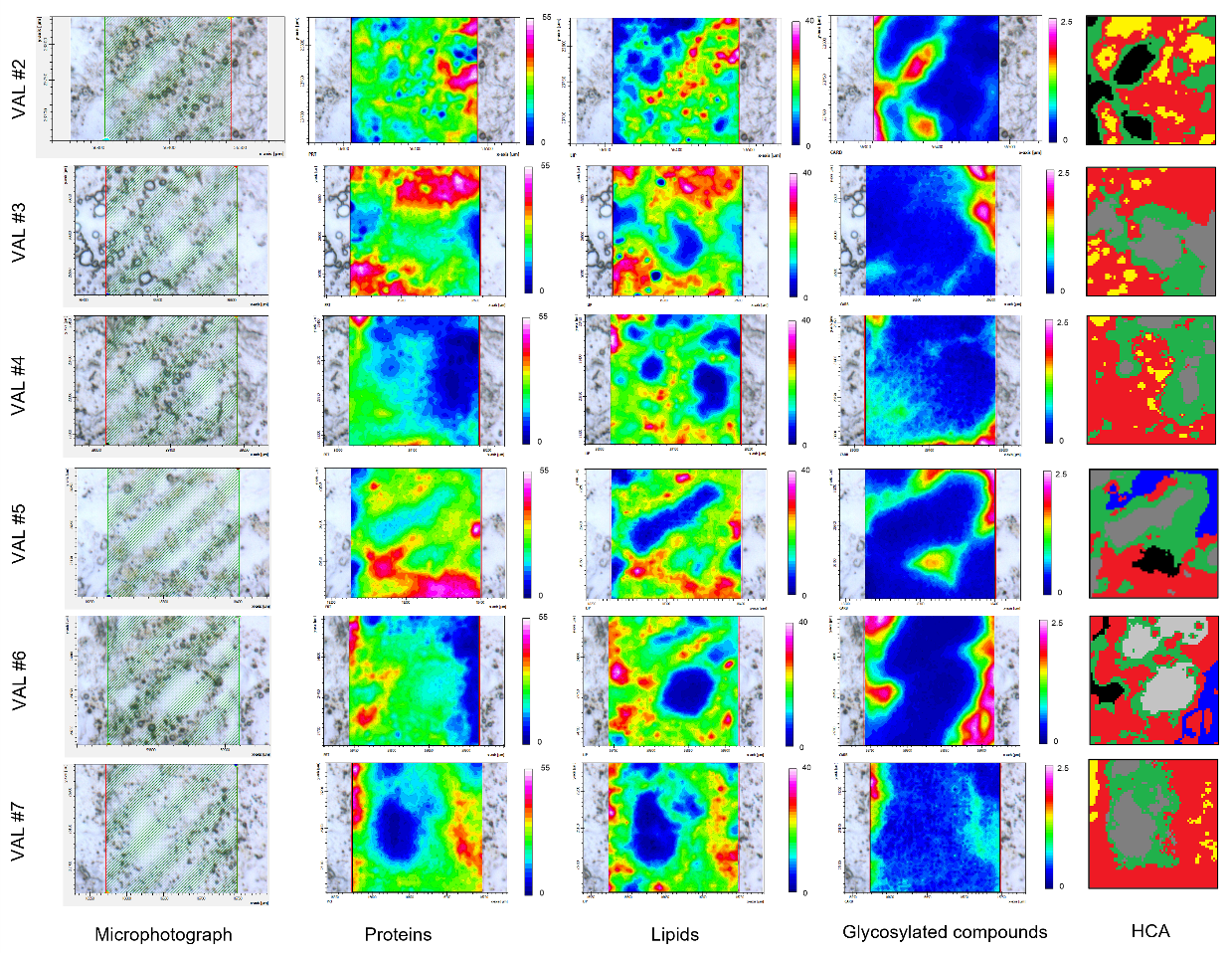


**Figure S7**

Black and white microphotographs, hyperspectral analysis and HCA cluster map of representative mussels digestive gland sections belonging to VAL group at 28 days. False colour imaging shows the topographical distribution of proteins, lipids and glycosylated compounds. The comparison between each black and white microphotograph with associated HCA cluster map allow to characterize main components of digestive tissue and to discriminate different histological areas of tubules, mainly the lumen from the epithelium. The spectral characterization (HCA) highlights: in grey scale semi-quantitative differences in terms of amount of glycosylated compounds present in the lumen (light grey corresponds to empty lumen, dark grey to medium, and black to higher levels of glycosylated compounds); in green the inner epithelium; in red the outer epithelium; in yellow the lipid-rich regions and in blue random spots of glycosylated compounds.


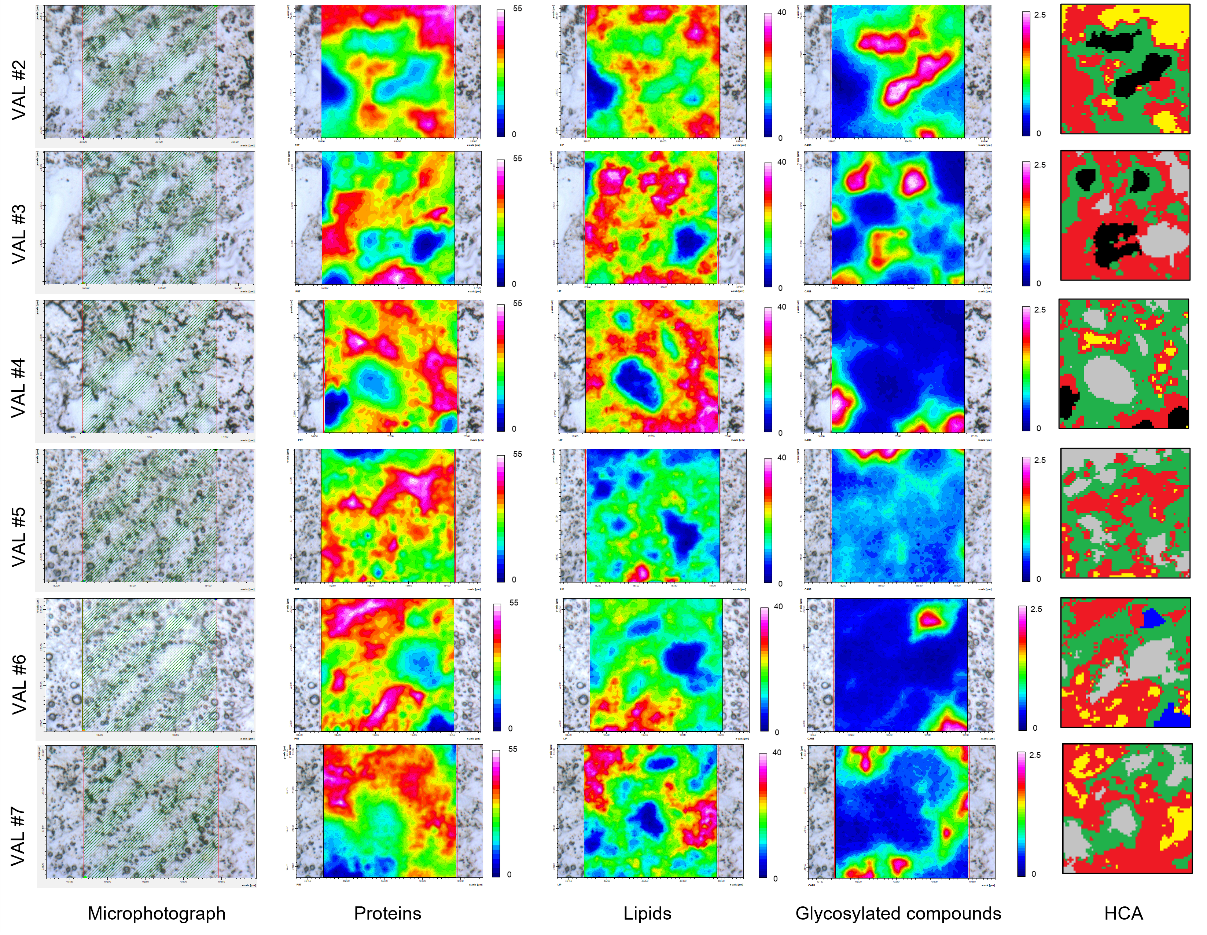


**Figure S8**

Black and white microphotographs, hyperspectral analysis and HCA cluster map of representative mussels digestive gland sections belonging to CBZ + VAL group at 14 days. False colour imaging shows the topographical distribution of proteins, lipids and glycosylated compounds. The comparison between each black and white microphotograph with associated HCA cluster map allow to characterize main components of digestive tissue and to discriminate different histological areas of tubules, mainly the lumen from the epithelium. The spectral characterization (HCA) highlights: in grey scale semi-quantitative differences in terms of amount of glycosylated compounds present in the lumen (light grey corresponds to empty lumen, dark grey to medium, and black to higher levels of glycosylated compounds); in green the inner epithelium; in red the outer epithelium; in yellow the lipid-rich regions and in blue random spots of glycosylated compounds.


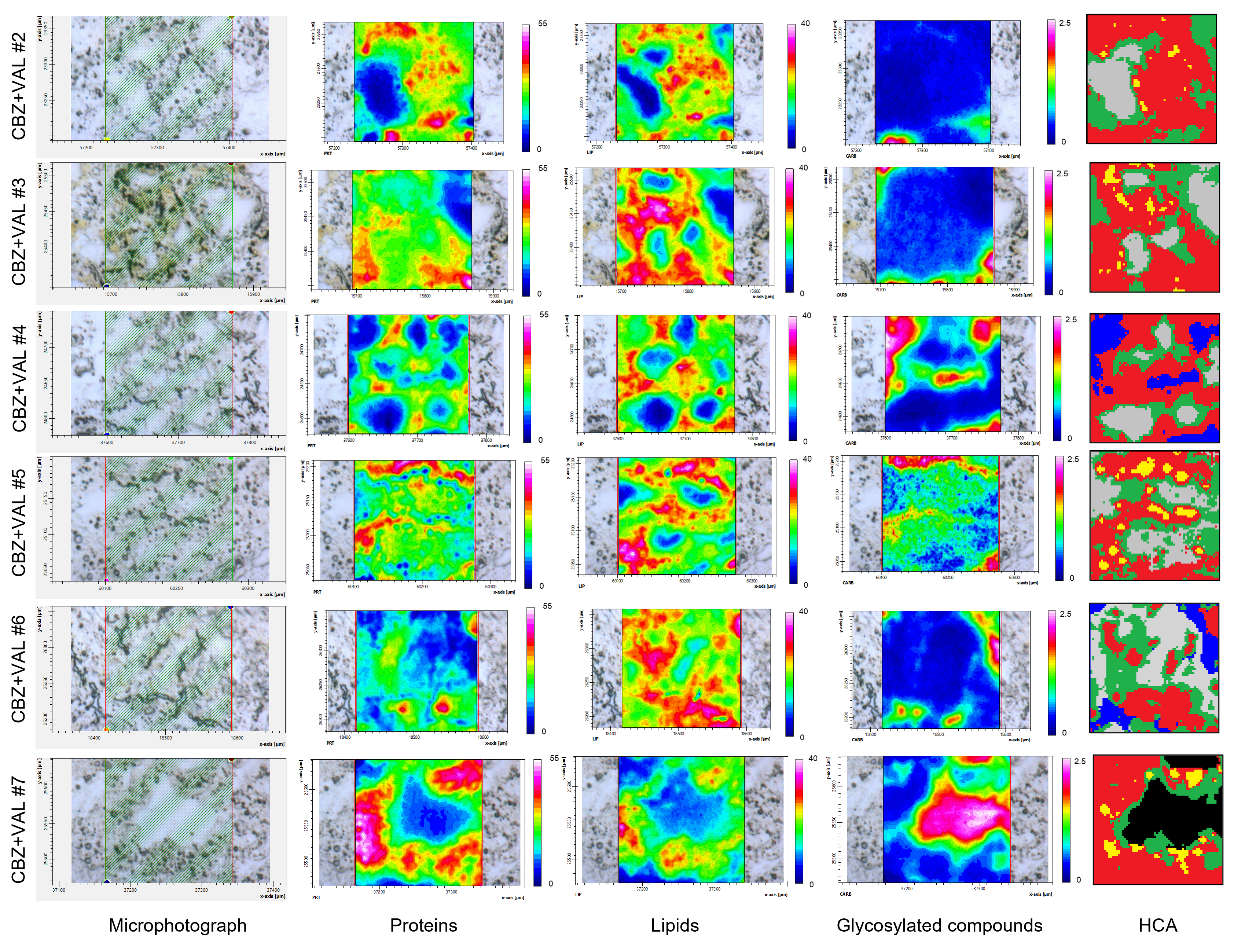


**Figure S9**

Black and white microphotographs, hyperspectral analysis and HCA cluster map of representative mussels digestive gland sections belonging to CBZ + VAL group at 28 days. False colour imaging shows the topographical distribution of proteins, lipids and glycosylated compounds. The comparison between each black and white microphotograph with associated HCA cluster map allow to characterize main components of digestive tissue and to discriminate different histological areas of tubules, mainly the lumen from the epithelium. The spectral characterization (HCA) highlights: in grey scale semi-quantitative differences in terms of amount of glycosylated compounds present in the lumen (light grey corresponds to empty lumen, dark grey to medium, and black to higher levels of glycosylated compounds); in green the inner epithelium; in red the outer epithelium; in yellow the lipid-rich regions and in blue random spots of glycosylated compounds.


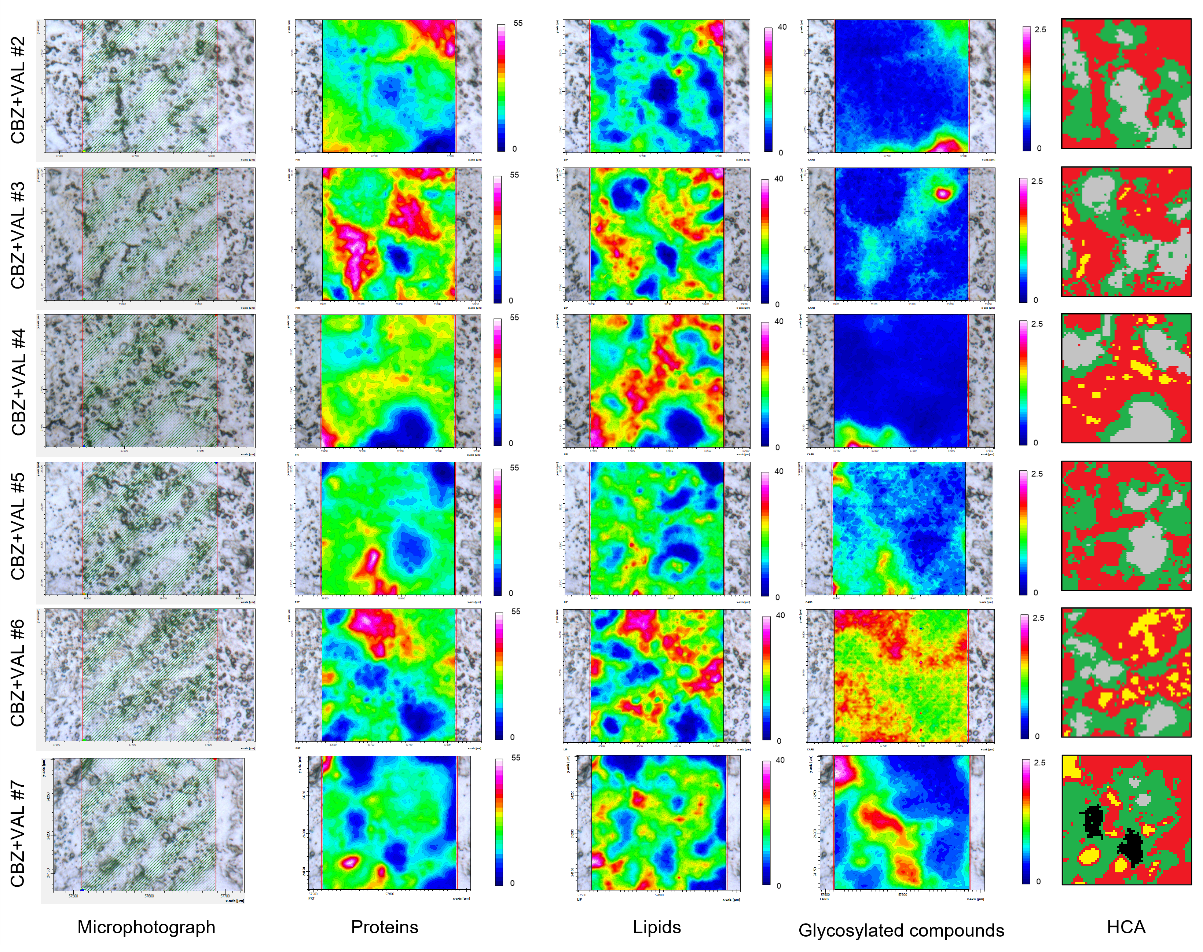

Supplement: Supplementary file 1 — Supplementary Information. [file 41598_2024_59663_MOESM1_ESM.docx]
